# Genetic Factors of Predisposition and Clinical Characteristics of Rheumatoid Arthritis in Russian Patients

**DOI:** 10.3390/jpm11060469

**Published:** 2021-05-25

**Authors:** Ekaterina A. Vetchinkina, Dmitry S. Mikhaylenko, Ekaterina B. Kuznetsova, Tatiana A. Deryagina, Ekaterina A. Alekseeva, Irina V. Bure, Andrey A. Zamyatnin, Marina V. Nemtsova

**Affiliations:** 1 Institute of Molecular Medicine, Sechenov First Moscow State Medical University (Sechenov University), 119991 Moscow, Russia; katevetchinkina@ya.ru (E.A.V.); dimserg@mail.ru (D.S.M.); kuznetsova.k@bk.ru (E.B.K.); ekater.alekseeva@gmail.com (E.A.A.); bureira@mail.ru (I.V.B.); nemtsova_m_v@mail.ru (M.V.N.); 2 Research Centre for Medical Genetics, 115522 Moscow, Russia; t.deryagina2018@yandex.ru; 3 Belozersky Institute of Physico-Chemical Biology, Lomonosov Moscow State University, 119991 Moscow, Russia; 4 Department of Biotechnology, Sirius University of Science and Technology, 1 Olympic Ave, 354340 Sochi, Russia

**Keywords:** rheumatoid arthritis, polymorphism, genetic predisposition, genotyping, NGS

## Abstract

Rheumatoid arthritis (RA) is a multifactorial disease caused by a genetic predisposition and environmental factors. Predisposing alleles of various genes have a relatively small influence on the disease risk when they appear separately, but in combination, they predispose an individual to RA development. We genotyped 125 patients with RA including 60 SNPs and sequenced coding part of six genes by next-generation sequencing (NGS) technology on a target panel (IAD177464_185). According to our data, the alleles HLA-DRB1*04, HLA-DRB1*01, HLA-B*27, *PTPN22* (rs2476601), *TNF* (rs1800629), *TPMT* (rs2842934), and *IL4* (rs2243250), and genotypes HLA-DRB1*04:04, HLA-DRB1*01:16, *PTPN22* (rs2476601), *TPMT* (rs2842934), were significantly associated with the RA development. Associations with clinical criteria (DAS28-CRP, HAQ-DI, and CDAI) and biochemical factors were investigated. We have shown that the *PADI4* genotypes (rs11203367, rs2240340, rs11203366, and rs874881) are significantly associated with the baseline levels of DAS28-CRP, HAQ-DI, and CDAI; genotypes *IL23R* (rs7530511) and *TNFRSF1A* (rs748004, rs2228144) with the level of anti citrullinated peptide antibodies (ACPA); the genotypes *DHODH* (rs3213422) and *MTHFR* (rs180113) with the concentration of C-reactive protein (CRP); and the genotypes *IL2RA* (rs2104286), *IRAK3* (rs11541076), and *IL4R* (rs1801275) with the level of rheumatoid factor (RF). Application of targeted NGS panel contributes to expanded genotyping to identify risk groups among the RA patients.

## 1. Introduction

Rheumatoid arthritis (RA) is a chronic systemic inflammatory disease associated with joint damage. RA is caused by autoimmune alterations that lead to erosive and destructive changes in connective tissues. RA is characterized by significant clinical polymorphism, progressive severe course, and serious complications, if not treated properly. According to the WHO, the incidence of the disease is 0.3–1% [1], with higher prevalence in women than in men by a ratio of 3:1, respectively [2].

Clinical and genealogical methods revealed a genetic predisposition to RA, so the disease is considered as multifactorial, arising from the interaction of unfavorable alleles of various genes and negative environmental factors [3]. The combination of several alleles associated with RA together either can result in a more severe course of the disease or become a protective factor. Currently, a contribution of molecular genetic factors to the development of RA is estimated as 50–60% [4]. Due to the high significance of hereditary factors, it remains relevant to create panels of genetic markers that reflect the risk of occurrence and characteristics of RA pathogenesis.

The literature describes more than 100 loci associated with RA, both for the human leukocyte antigens (HLA) and non-HLA genes [5]. HLA is a system of human leukocyte antigens that represents antigens for recognition by T cells. HLA genes are localized in 6p21.3, and they include nearly 60% of polymorphic genetic variants of predisposition to RA. Besides, their associations with various clinical and pharmacogenetic characteristics are also shown [6].

RA is common in all populations and ethnic groups. The genome-wide association study (GWAS) has demonstrated that the genes of predisposition to RA may differ in patients of different ethnic groups [7,8]. The genetic factors of RA in the Russian population have not been sufficiently studied. Several studies have been published that consider individual polymorphic variants, as well as data on different Slavic groups [9,10].

The allele (genotype) frequency in gnomAD comprehensively reflects the genetic structure of Caucasian populations in general, as well as that of the countries of Western and Northern Europe and of some countries of Eastern Europe. However, the Eastern European populations living in the countries of the former Soviet Union (Belarus, the European part of Russia, Ukraine, and Moldova) remain insufficiently characterized. The study is also aimed at searching for allelic (genotypic) associations with RA that are specific for the Russian population.

The identification of genetic factors that affect the clinical course before disease manifestation will allow one to propose preventive recommendations, as well as to predict the dynamics of the clinical picture and further conduct effective targeted therapy with novel drugs. Using a targeted NGS panel, we analyzed known loci and candidate genes to reveal associations with the pathogenesis, clinical course, and biochemical characteristics of RA in Russian patients.

## 2. Materials and Methods

### 2.1. Ethical Approval and Patients

The study involved 125 RA patients from European part of Russia, over the age of 18, with the mean age of 50.4 years. The number of women was significantly higher than men (109 and 16, respectively). The median duration of the disease was 5.91 years. The majority of patients (90.4%) had high disease activity according to the DAS28, CDAI, and HAQ-DI scales: 1—low (2.6 < DAS28 < 3.2), 2—medium (DAS28 3.2—5.1), and 3—high (DAS28 > 5.1). The initial mean value of DAS28 was 5.94, CDAI—39.43, and HAQ-DI—1.69. The initial level of C-reactive protein (CRP) was 21.0 mg/L, anti-cyclic citrullinated peptide antibodies (anti-CCP)—664.35 IU/mL, and rheumatoid factor (RF)—192.2 IU/mL. The level of anti-CCP >10 IU/mL was observed in 80.8% patients, and the level of RF ≥ 15 IU/mL in 84.0% of patients. The data are presented in the Table 1. The main exclusion criteria were: patients with any other inflammatory or systemic diseases, pregnant or lactating women, and patients treated with drugs that targeting IL6 or IL6R.

The current study was performed on DNA samples from the peripheral blood of Russian patients. All subjects gave their informed consent for participation in the study and publication of the results. The study was conducted in accordance with the Declaration of Helsinki, and the protocol was approved by the Ethics Committee of Sechenov First Moscow State Medical University (Sechenov University), 13 July 2016.

### 2.2. DNA Isolation and Genotyping HLA and Non-HLA Genes

DNA from peripheral blood leukocytes of patients was extracted using the kit “ExtractDNA Blood and Cells” (Eurogen, Russia).

The HLA system was genotyped using the «HLA-DNA-TEX» reagent kits for typing the *DRB1* gene and «HLA-B27» (DNA-Technology, Russia) according to the manufacturer’s protocols.

Polymorphic variants localized in non-HLA genes were genotyped using the AmpliSeq panel IAD177464_185 (Thermo Fisher Scientific, Waltham, MA, USA). This multigenic panel includes coding sequences of the *IL6, IL6R, TNFRSF1A, CTLA4, IL10, IL23R, PADI4,* and partially HLA-DRB1 and HLA-B genes, as well as 60 short genome fragments with polymorphic variants (mainly in the coding regions of genes) associated with the development, activity of clinical manifestations, and effectiveness of treatment of patients with RA according to the literature data. A complete list of the analyzed sequences is available in the [11].

The libraries were prepared using the Ion AmpliSeq Library Kit 2.0 (Thermo Fisher Scientific) according to the manufacturer’s protocols. The preparation of 540 chips was carried out at the Ion Chef station, followed by semiconductor sequencing on the IonS5 Systems device (Thermo Fisher Scientific, USA). Genetic variants were annotated with ANNOVAR software. Visual data analysis, manual filtering of sequencing artefacts, and sequence alignment were performed using the integrative genomics viewer (IGV).

### 2.3. Statistical Analysis

Statistical analysis of the frequency distribution of alleles and genotypes of patient groups and controls was performed using MS Excel. For HLA-DRB1 and HLA-B27, a comparison was performed for the HLA allele frequencies in a Russian population, calculated based on the total number of alleles and the number of variant alleles from the allele frequency net database (AFND) [12]. For non-HLA genes, allele and genotype frequencies in RA patients were compared with the population frequencies in the Caucasian population, calculated based on the total number of alleles and the number of variant alleles from the Genome Aggregation Database (gnomAD) [13].

For dichotomous variables, the exact Fisher test or the Mann–Whitney test were used; the Chi-square test or the nonparametric Kruskal–Wallis test were used when comparing categorical indicators of three or more independent samples; for all genotypes, the Chi–square test was used for categorical indicators, and ANOVA and Student’s t–test were used for pairwise comparisons. For continuous variables, the Kruskal–Wallis test and the Wilcoxon–Mann–Whitney test were used, respectively. The relative risk of RA development was assessed using odds ratio (OR) with a 95% confidence interval (CI). Multiple hypotheses were corrected using the Benjamin-Hochberg method (FDR test). Statistical analysis was carried out using the statistical program STATISTICA version 13.5.0. *p*-Values < 0.05 were considered statistically significant for all comparisons.

## 3. Results

### 3.1. Identification of Genetic Factors of Predisposition to RA

The allele frequency and genotype distribution associated with predisposition to RA were estimated in Russian patient samples and control samples. Statistically significant differences were observed for polymorphic gene loci: *TNF* [rs1800629]; *FCGR2A* [rs1801274]; *IL4* [rs2243250]; *IL17A* [rs2275913]; *PTPN22* [rs2476601]; *TPMT* [rs2842934]; *BTNL2* [rs3817963]; *STAT4* [rs7574865]; HLA-B allele 27; groups of HLA-DRB1 *1 and *4 alleles; and genotypes *01:04, *01:16, *04:04, *04:16. The results are presented in Table 2.

The highest risk of RA among alleles of the HLA-DRB1 was associated with the allele *01 OR = 4.7 [CI: 3.3–6.8], *p* = 0.0004 and *04 OR= 3.1 [CI: 2.3–4.26], *p* = 0.00001. Among the genotypes of the HLA-DRB1, the highest risk was shown by the genotype *04:04 OR = 13.4 [CI: 6.4–27.9], *p* = 0.00001 and *01:16 OR = 8.3 [CI: 3.53–19.55], *p* = 0.00001. Genotype *04:16 had an OR value of 4.3 [CI: 1.6–11.7], *p* = 0.007, and in the additive interaction model in combination with the HLA-B*27 allele, OR = 9.6 [CI: 1.8–51.2], *p* = 0.012. Genotype *01:03 was close to the significance level with OR = 2.7 [CI: 1.05–7.1], *p* = 0.05, but in the additive interaction model in combination with HLA-B*27 allele demonstrated a significant association OR = 14.5 [CI: 2.27–92.6], *p* = 0.004. The HLA-B*27 allele is associated with the risk of RA OR = 2.2 [CI: 1.37–3.7], *p* = 0.001. Protective properties that reduce the risk of RA were found for alleles HLA-DRB1: *07 (OR = 0.6 [CI: 0.4–0.9], *p* = 0.02), *08 (OR = N/A, *p* = 0.0076), and *13 (OR = 0.5 [CI: 0.3–0.89], *p* = 0.01).

High-risk RA polymorphisms in non-HLA genes are: *PTPN22* [rs2476601] allele T OR = 2.3 [CI: 1.7–3.2], *p* = 0.0001; *TNF* [rs1800629] allele G OR = 2.25 [CI: 1.4–3.5], *p* = 0.0001; *TPMT* [rs2842934] allele G OR = 1.55 [CI: 1.2–2], *p* = 0.002; *IL4* [rs2243250] allele T OR = 1.6 [CI: 1.2–2.1], *p* = 0.0025, etc. The highest OR values among the genotypes of non-HLA genes are shown for the homozygous TT genotype of the *PTPN22* gene OR = 8.5 [CI: 4.6–15.56], *p* = 0.001 and the GG genotype of *TPMT* OR = 2.5 [CI: 1.4–4.5], *p* = 0.0016.

Protective properties among alleles of non-HLA genes were revealed for polymorphisms: *PTPN22* [rs2476601] allele C OR = 0.4 [CI: 0.3–0.57], *p* = 0.0001; *TNF* [rs1800629] allele A OR = 0.4 [CI: 0.3–0.7], *p* = 0.0001; *TPMT* [rs2842934] allele A OR = 0.64 [CI: 0.5–0.85], *p* = 0.002; and *IL4* [rs2243250] allele C OR = 0.63 [CI: 0.47–0.84], *p* = 0.0025. Protective properties among the genotypes of non-HLA genes were revealed for *PTPN22* genotype CC OR = 0.5 [CI: 0.3–0.7], *p* = 0.001; *TPMT* genotype AA OR = 0.64 [CI: 0.45–0.92], *p* = 0.0016, etc.

The correction of multiple hypotheses was carried out using the Benjamin-Hochberg method (FDR test); the adjusted *p*-values are presented in Table 3. After the correction, the HLA-DRB1*04, HLA-DRB1*01, and HLA-B*27 alleles remained significant, as well as *PTPN22* [rs2476601], *TNF* [rs1800629], *TPMT* [rs2842934], and *IL4* [rs2243250] for non-HLA genes.

### 3.2. Genetic Factors Associated with the Clinical Course of the Disease

Analysis of the SNPs genotype frequencies and the initial clinical characteristics of the patients in our sample revealed associations for a number of genes and loci. However, when adjusting for multiple hypothesis testing, no significant associations were revealed. The table presents the loci and genes with significant associations with the initial clinical and biochemical parameters, but no significant associations were found for the other loci (Supplementary Material Table S1).

The study of correlations of genotype frequencies with clinical characteristics and biochemical parameters of the patients revealed significant associations mainly for non-HLA genes. The HLA-B27 genotypes were significantly associated only with elevated anti-CCP level (*p* = 0.008), while *PADI4* [rs11203367, rs2240340, rs11203366, rs874881], *AMPD1* [rs17602729], *IL23R* [rs10889671, rs11465770], *IL4R* [rs1801275], *IL4* [rs2243250], and *IL6* [rs2069849] were significantly associated with the severity of the disease (DAS28, CDAI, HAQ-DI). *MTHFR* [rs180113] and *DHODH* [rs3213422] were associated with *CRP* level; *IRAK3* [rs11541076], *IL2RA* [rs2104286], and *IL4R* [rs1801275] were associated with the rheumatoid factor (RF) level; and *IL23R* [rs7539625, rs7530511, rs10889671], *IL2RB* [rs3218253], *IL6R* [rs 2228144], *STAT4* [rs7574865], and *TNFRSF1A* [rs767455, rs1800693] were associated with anti-CCP level (Table 4).

**Figure 1:**
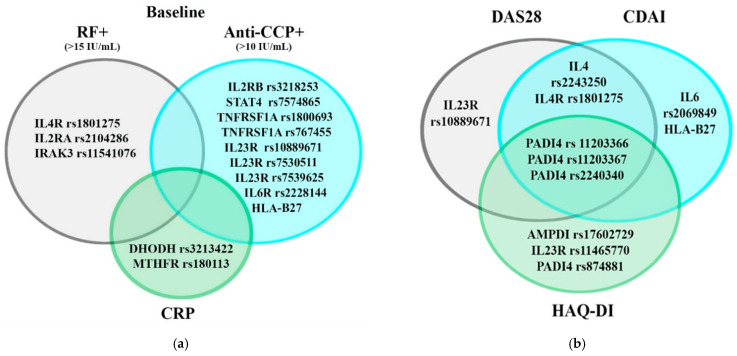
Venn diagrams, demonstrating the associations of polymorphic variants and biochemical (**a**) and clinical (**b**) characteristics of patients.

To demonstrate polymorphisms associated with several characteristics, Venn diagrams were constructed (Figure 1). Thus, we identified the loci *PADI4* [rs11203367, rs2240340, rs11203366], *IL4R* [rs1801275], and *IL4* [rs2243250], which are associated with the severity of clinical manifestations by several clinical criteria.

## 4. Discussion

Predictive medicine, which makes it possible to assess the development and course of the disease, requires the study of not only clinical indices, biochemical parameters, and individual characteristics of patients but also, first of all, the genetic predisposition to its development and the severity of the clinical course. Today, there are many studies investigating the genetic factors of predisposition to RA [3,5,14]. However, their results are obtained in different populations and samples and therefore are ambiguous.

In numerous studies, the carriage of some alleles of the major histocompatibility complex class II HLA-DR1 was significantly associated with the risk of RA. In our study, the HLA-DRB1*01 and *04 alleles demonstrated predisposition to RA: OR = 4.7 [CI: 3.3–6.8], *p* = 0.0004 and OR = 3.1 [CI: 2.3–4.3], *p* = 0.00001, respectively. These results are in concordance with those obtained by Guseva I.A. et al. in study conducted in Russian patients. However, only *07 was found in both studies as a protective allele, while other alleles differed, namely, *08 and *13 in our study and *02 and *06 in Guseva’s study. Our patients also had the most common genotype *04:04—11.4%, whereas in the study of Guseva et al.—*01:01—4.2% [15]. The variations of the results are probably due to the investigation of early RA, whereas our sample of patients was in the older age group.

Kuranov et al. performed a meta-analysis of the HLA-DRB1 associations with RA development. The risk varied depending on the ethnicity of the population: the highest risk was determined HLA-DRB1*04 among the Western Europe residents for the British OR = 7.02, whereas the lowest was in Asians for Pakistanis OR = 0.65, with the allele HLA-DRB1*04 being a risk factor for RA, and HLA-DRB1*13 being a protector [16]. HLA-DRB1*04:01, *04:04, and *04:08 are associated with RA in Europeans, and HLA-DRB1*04:05 with RA in Asian populations [17]. In our group, the association of the HLA-DRB1*04 allele and the DRB1*04:04 genotype with the development of RA are in concordance with the abovementioned studies, whereas the data obtained on the HLA-DRB1*01 alleles and the HLA-DRB1*01*16 genotype were different, which could be explained by the population characteristics.

The investigation of Portuguese patients also identified the HLA-DRB1*01 (OR = 1.79) and HLA-DRB1*04 (OR = 2.81) polymorphisms as predisposing one to RA, and the HLA-DRB1*13 allele as protective [18]. The protective effect of the HLA-DRB1*13 allele (OR = 0.54 [CI 0.38–0.77]), as in our study, was shown in a meta-analysis of four European populations. The authors concluded that the frequency of occurrence and negative association of the HLA-DRB1*13 allele varied from north to south, with an increase in OR in the Nordic countries [19].

For non-HLA genes, we found a significant effect of the TT genotype and the *PTPN22* T allele [rs2476601] on the risk of RA development: (TT) OR = 8.5 [CI: 4.6–15.56], *p* = 0.001, (T) OR = 2.3 [CI: 1.7—3.2], *p* = 0.0001. The *PTPN22* encodes lymphoid protein tyrosine phosphatase (LYP), which is a negative regulator of the T-cell receptor-mediated signaling pathway (TCR) and prevents spontaneous activation of T cells. The non-synonymous substitution of rs2476601 changes arginine (R) at position 620 to tryptophan (W), which affects the physical binding to Csk tyrosine kinase during T-cell activation [20]. The association of *PTPN22* polymorphism [rs2476601] with the development of RA was found in different populations. The association of the T allele with the development of RA has been shown in Europeans and representatives of other races, in the study of a Hungarian sample of patients, as well as in patients of the United Kingdom. The meta-analysis from 2020 reports that *PTPN22* [rs2476601] increases the risk of RA in Europeans and Africans [21].

A characteristic of the *PTPN22* polymorphism [rs2476601] is the variations of the allele frequencies in different populations. In European populations, there is an increase in the T allele frequency from south to north. Its frequency in the population of Italy and Sardinia is 2–3%, in the population of Western Europe—7–8%, and more than 10% in the population of Scandinavia. Therefore, in some Southern European populations, the association of *PTPN22* gene polymorphism with RA is weaker or absent [22]. In Latvian residents, the T allele is associated with the risk of RA development OR = 1.96 [CI: 1.5–2.5], *p* = 0.000006 and with RF-positive RA: OR = 2.38 [CI: 1.60–3.54, *p* = 0.0001] [23]. Song et al. performed a meta-analysis and confirmed that the T allele is associated with the risk of RA development in different populations [24].

We found a 1.4-fold increase in the risk of RA [CI: 1.04–1.83], *p* = 0.025 for the *STAT4* polymorphism [rs7574865] in the presence of a minor T allele. It is known that the presence of the T allele increases the expression of *STAT4* mRNA and protein, which stimulates and activates cytokine-related signaling pathways [25]. The minor T allele creates a high risk of developing autoimmune disorders. It is associated with an increase in the level of IFN-α, which is indicated for patients with systemic lupus erythematosus (SLE) [26]. A study of RA patients in Slovakia revealed statistically significant associations with RA risk for *STAT4* and some other genes (HLA-DRB1*04, *PTPN22*) [27].

Yongshuai Jiang and co-authors performed a meta-analysis of 125 SNPs related to RA and demonstrated that *PTPN22* [rs2476601] and STAT4 [rs7574865] are significantly associated with the RA development, which is in concordance with our results [28]. Fcγ receptor 2A (FCGR2A) is expressed in the most types of immunocomponent cells and plays an important role in the regulation of many immunological and inflammatory processes. We found that the *FCGR2A* [rs1801274] allele T increases the risk of RA in 1.4 times [CI: 1.08–1.8], *p* = 0.011, and the protective allele G reduces the risk of RA: OR = 0.72 [CI: 0.56–0.93], *p* = 0.011. A meta-analysis performed by Lee et al. revealed an association of the protective allele G of the *FCGR2A* with RA in Europeans OR = 0.8 [CI: 0.687–0.968], *p* = 0.02, but not in Asian population: OR = 0.9 [CI: 0.778–1.040] *p* = 0.15 [29]. Kyogoku et al. studied the polymorphisms of the *FCGR2B, FCGR2A, FCGR3A*, and *FCGR3B* in Japanese population and found no significant association with RA [30]. Due to the small number of studies of the FCGR2A and the small sample sizes, this gene remains insufficiently characterized.

In our study, we demonstrated that the predisposition to RA in Russian patients is determined mainly by the same genes and polymorphic loci, as in Eastern Europe residents.

Genetic factors can influence not only the risk of development, but also the severity of the clinical course of RA, and therefore serve as diagnostic and prognostic markers. Identification of genetic markers associated with the RA prognosis is often more challenging than identification of predisposition markers. This is because the course and outcome of the disease depends not only on genetic factors but also on epigenetic changes, as well as on individual characteristics of the response to therapy and concomitant diseases.

In the current study, we showed that *PADI4* (rs11203367, rs2240340, and rs11203366), *IL4R* (rs1801275), and *IL4* (rs2243250) are associated with the RA clinical characteristics according to the criteria DAS28, CDAI, and HAQ. In some cases, genetic factors that are responsible for the predisposition to RA development are associated with its clinical course.

The *PADI4* encodes peptidyl-arginine deaminase, and this enzyme converts arginine amino acid residues to citrulline residues. Deregulation of this process can contribute to citrulline antibodies formation. *PADI4* is localized in the region 1p36 and associated with RA development in Asian population. Substitutions in *PADI4* in codons G55S [rs11203366], V82A [rs11203367], and G112A [rs874881] reduce the mRNA stability of this gene. According to the meta-analysis, where about 20 thousand RA patients and more than 25 thousand controls were analyzed, polymorphism -94G/A is associated with RA in Asian populations, -92C/G—in African, and -90C/T in Latin American [31].

The carriage of genotypes associated with a decreased activity of *PADI4* could be exacerbated by the condition of the patient microbiota or by a previous infectious disease, when the bacteria additionally produce similar targets for citrullination and even have the ability to auto-citrullinate [32]. Deregulation of citrullination contributes to an increase of anti-CCP level and a more severe course of the disease, estimated by the clinical indices. In our study, certain *PADI4* genotypes (rs11203367, rs2240340, and rs11203366) were associated with higher baseline values of the clinical indices DAS28, CDAI, and HAQ-DI. Presumably, an analysis of a microbiota condition in patients in combination with genetic factors could be a perspective method for estimation of predisposition to RA.

The *IL4* induces the differentiation of naïve T helper cells into Th2 cells and thus affects the development and severity of RA. It fulfils biological activity by binding to the receptor IL4R and stimulating target cell growth by activating transcription and IgE production. Our study revealed that the polymorphic variant of the *IL4* [rs2243250] (T allele) is associated with the initial value of the disease activity according to DAS28 (*p* = 0.015), with the initial value of the clinical CDAI index (*p* = 0.014) and the risk of developing RA: OR = 1.6 [CI: 1.2–2.1], *p* = 0.0025. Conversely, allele C plays a protective role: OR = 0.61 [0.43–0.87], *p* = 0.0025. In the study of the Polish population, the distribution of IL4 genotypes in RA patients was similar to that in the control. However, the active form of RA was more often diagnosed in patients with the T allele (CT and TT genotypes) when compared to the homozygous CC genotype. Besides, the disease activity indicators according to DAS28 were significantly increased in T allele carriers [33]. A study in Egypt showed the association of the IL4 polymorphism T allele rs2243250 with RA development (0.03) and the severity of the disease (<0.001), as well as the IL4R polymorphism rs1801275 with RA severity (<0.001) [34]. Carriers of the IL4R allele A1902 rs1801275 were significantly more often RF-positive and had a severe form of RA [35]. SE- and RF-positive African Americans with RA had a higher risk of rheumatoid nodules development in the presence of the AA and AG genotypes of the *IL4R* polymorphism rs1801275 (OR = 8.45 [CI: 1.57–45.44], *p* = 0.01 and OR = 3.57 [CI: 1.18–10.76], *p* = 0.02, respectively) [36]. However, when studying the role of polymorphic variants in the *IL4* and *IL4R* in RA patients of the Caucasian race, no correlation was found with the predisposition to RA and the disease severity [37].

We were unable to identify polymorphic variants that are associated with changes in the overall expression of all three biochemical parameters (RF, anti-CCP, and CRP) that determine the manifestation of a more severe form of seropositive RA. However, we identified loci that were significantly associated with each of these biochemical parameters.

Rheumatoid factor (RF) is an antibody that recognizes the Fc conservative part of IgM. They are detected in 60–80% RA patients, and their presence determines the more severe course of the disease and early destructive joints damage [38]. RF is not specific for RA, and occurs in patients with other autoimmune diseases as well [39], whereas anti-CCP are more specific. In the study of Japanese patients, it was shown that the expression of anti-CCP correlates with RF expression. Thus, more than 90% of patients with anti-CCP-positive RA were also RF-positive [40].

We found a statistically significant association of *IL2RA* [rs2104286], *IRAK3* [rs11541076], and *IL4R* [rs1801275] with RF-positive RA. Other authors have published data that patients from south-eastern and eastern France have a risk of developing anti-CCP-positive RA in the presence of the HLA-DRB1 *04 and *01 alleles [41]. High RF titers were detected in carriers of the T allele of the IL4 in Egyptian residents, who had a more severe disease in comparison with C allele carriers [34].

An investigation of 125 RA patients may be not sufficient to identify all the existing differences of the polymorphism frequencies, associated with the clinical course of the disease. Nevertheless, the most pronounced associations with a high probability ratio were identified even in such a limited sample size. It is important for improving the understanding of the genetic predisposition to RA in the Russian population and may encourage further larger-scale studies.

The aim of genetic studies of RA is to predict the predisposition and severity of the disease and find an appropriate treatment for a patient. The genetic heterogeneity of RA can be a serious obstacle in the clinical application of genetic data, and large-scale studies are required to predict the course of the disease and a therapy response. RA patients with genotypes that contribute to the higher RA severity are likely to experience rapid disease progression. Thus, the increased use of therapeutic agents may be more prominent for them. The identification of genetic marker systems that can predict the response to treatment is an important step to the personalized medicine. Application of such systems will help to avoid side effects and decrease costs. However, the application of genetic factors to predict RA severity and a drug response should be developed very carefully, integrating them into a system and considering the ethnic characteristics of the patient sample.

The use of next-generation sequencing will provide a comprehensive approach to genotyping clinical groups of patients with various rheumatic diseases [42]. Using the knowledge of genomics and epigenomics about the mechanisms of disease progression (aberrations in chromatin or DNA methylation, and microRNA pattern) and the influence of environmental factors and microorganisms that promote the cross-reactions with anti-CCP and chronic inflammation will explain the resistance of some patients to therapy with certain drugs. The developed targeted NGS panels will allow identification of therapeutic targets and support a personalized approach in medicine to diagnosis and effective RA treatment.

## 5. Conclusions

The obtained data indicate the contribution of the candidate genes identified by us to the RA pathogenesis. We identified polymorphic variants whose alleles and genotypes are associated with gender, age, duration of the disease, baseline levels of CRP, anti-CCP, and RF, as well as the initial clinical characteristics of patients, namely, criteria CDAI, DAS28-CRP, and HAQ-DI. These results will contribute to the identification of RA risk groups among the Russian population and groups of patients with a more severe course of the disease, for which it may be necessary to optimize the monitoring and treatment protocols.

## Figures and Tables

**Table 1:** Characteristics of the RA patients.

Clinical Characteristic	RA (N = 125)
Age (years)	
mean ± SD	50.4 ± 13.14
[Min, max]	[22, 82]
Gender	
Female	109 (87.2%)
Male	16 (12.8%)
Weight (kg)	
mean ± SD	73.63 ± 16.366
[Min, max]	[41.0, 131.0]
Disease duration (years)	5.91
Min, max	[2.61, 11.16]
Disease severity	
Moderate (DAS28-CRP > 3.2 to ≤5.1)	10 (8.0%)
High (DAS28-CRP > 5.1)	113 (90.4%)
DAS28-CRP	
mean ± SD	5.94 ± 0.643
[Min, max]	[4.5, 8.1]
CDAI	
mean ± SD	39.43 ± 8.701
[Min, max]	[24.8, 69.3]
HAQ-DI	
mean ± SD	1.6890 ± 0.4998
[Min, max]	[0.125, 2.875]
CRP (mg/mL)	
mean ± SD	21.0 ± 20.83
[Min, max]	[1, 120]
Anti-CCP (IU/mL)	
mean ± SD	664.35 ± 999.308
[Min, max]	[0.4, 6044.8]
RF (IU/mL)	
mean ± SD	192.2 ± 240.95
[Min, max]	[7, 1540]
Anti-CCP (>10 IU/mL)	
Yes	101 (80.8%)
No	22 (17.6%)
RF (≥15 IU/mL)	
Yes	105 (84.0%)
No	20 (16.0%)
Basal Anti-CCP and RF	
Low	25 (20.0%)
Medium	81 (64.8%)
High	17 (13.6%)

**Table 2:** Polymorphic gene loci with revealed statistically significant differences in the frequencies of alleles and genotypes of polymorphic variants in the studied sample of RA patients in comparison with the population frequencies.

Non-HLA	Frequency of Alleles and Genotypes		*p*	OR [CI 95%]
Alleles and Genotypes	(abs. Value/Frequency)			
	RA (*n* = 125)	Control (GnomAD)		
*PTPN22* (rs2476601)				
Allele C	192/0.77	13661/0.89	0.0001	0.4 [0.3–0.57]
Allele T	58/0.23	1753/0.11		2.3 [1.7–3.2]
CC	80/0.64	6058/0.79	0.001	0.5 [0.3–0.7]
CT	32/0.26	1545/0.20		1.37 [0.9–2]
TT	13/0.1	104/0.013		8.5 [4.6–15.56]
*FCGR2A* (rs1801274)				
Allele G	105/0.42	7710/0.5	0.011	0.72 [0.56–0.93]
Allele A	145/0.58	7668/0.5		1.4 [1.08–1.8]
GG	19/0.15	1959/0.255	0.025	0.52 [0.32–0.86]
GA	67/0.54	3792/0.493		1.2 [0.83–1.7]
AA	39/0.31	1938/0.252		1.3 [0.92–1.97]
*STAT4* (rs7574865)			0.025	
Allele G	181/0.72	12071/0.78		0.72 [0.55–0.96]
Allele T	69/0.28	3331/0.22		1.4 [1.04–1.83]
			0.068	
GG	64/0.52	4717/0.62		0.66 [0.5–0.94]
GT	53/0.42	2637/0.34		1.4 [0.99–2]
TT	8/0.06	347/0.04		1.45 [0.7–3]
*IL4* (rs2243250)			0.0025	
Allele T	63/0.25	2705/0.18		1.6 [1.2–2.1]
Allele C	187/0.75	12699/0.82		0.63 [0.47–0.84]
			0.002	
TT	9/0.07	265/0.0344		2.2 [1.1–4.3]
CT	45/0.36	2175/0.2824		1.43 [0.99–2.07]
CC	71/0.57	5262/0.6832		0.61 [0.43–0.87]
*IL17A* (rs2275913)			0.034	
Allele G	143/0.57	9822/0.64		0.76 [0.59–0.97]
Allele A	107/0.43	5574/0.36		1.32 [1.02–1.7]
			0.015	
GG	45/0.36	3113/0.40		0.83 [0.57–1.2]
GA	53/0.42	3596/0.47		0.84 [0.59–1.2]
AA	27/0.22	989/0.13		1.87 [1.2–2.88]
*TPMT* (rs2842934)			0.002	
Allele A	175/0.7	12074/0.78		0.64 [0.5–0.85]
Allele G	75/0.3	3330/0.22		1.55 [1.2–2]
			0.0016	
AA	63/0.5	4714/0.61		0.64 [0.45–0.92]
AG	49/0.39	2646/0.34		1.23 [0.86–1.77]
GG	13/0.1	342/0.04		2.5 [1.4–4.5]
*TNF* (rs1800629)			0.0001	
Allele A	21/0.08	2636/0.17		0.4 [0.3–0.7]
Allele G	229/0.92	12780/0.83		2.25 [1.4–3.5]
			N/A	
AA	0/0	257/0.033		0 [0]
GA	21/0.17	2122/0.275		0.53 [0.33–0.85]
GG	104/0.83	5329/0.691		2.2 [1.4–3.54]
*BTNL2* (rs3817963)			0.017	
Allele C	82/0.33	4001/0.26		1.4 [1.06–1.8]
Allele T	168/0.67	11387/0.74		0.72 [0.55–0.94]
			0.015	
CC	17/0.14	544/0.07		2 [1.23–3.5]
CT	48/0.38	2913/0.38		1.02 [0.7–1.5]
TT	60/0.48	4237/0.55		0.75 [0.5–1.07]
HLA-alleles and genotypes	RA (*n* = 114)	Control (AFND)	*p*	OR [CI 95%]
HLA-DRB1				
Allele *01	45/19.74	474/11.85	0.0004	4.7 [3.3–6.8]
Allele *04	62/27.19	427/10.675	0.00001	3.1 [2.3–4.26]
Allele *07	20/8.77	562/14.05	0.02	0.6 [0.4–0.9]
Allele *08	0/0	139/3.475	N/A	N/A
Allele *13	18/7.89	548/13.7	0.01	0.5 [0.3–0.89]
*01:03	5/4.39	33/1.65	0.05	2.7 [1.05–7.1]
*01:04	7/6.14	49/2.45	0.017	2.6 [1.15–5.9]
*01:16	8/7.02	18/0.9	0.00001	8.3 [3.53–19.55]
*04:04	13/11.4	19/0.95	0.00001	13.4 [6.4–27.9]
*04:16	5/4.39	21/1.05	0.007	4.3 [1.6–11.7]
HLA-B27			0.001	
B27+	21/17.8	176/8.8		2.2 [1.37–3.7]
B27−	97/82.2	1824/91.2		0.45 [0.27–0.73]

GnomAD—Genome Aggregation Database; AFND—allele frequency net database; *p*-value—probability of the null hypothesis; OR—odds ratio; CI—confidence interval. N—the number of patients in the sample. The *p*-value is shown for comparing allele frequencies in the study population with the control using the Chi-square test and the Fisher exact test.

**Table 3:** Adjusted values, obtained using the Benjamin-Hochberg method.

Alleles	*p*	Adj. *p*	Genotypes	*p*	Adj. *p*
HLA-DRB1*04	0.00001	0.0008	HLA-DRB1*04:04	0.00001	0.001
*PTPN22*(rs2476601)	0.0001	0.004	HLA-DRB1*01:16	0.00001	0.001
*TNF* (rs1800629)	0.0001	0.003	*PTPN22*(rs2476601)	0.001	0.038
HLA-DRB1*01	0.0004	0.008	*TPMT* (rs2842934)	0.002	0.046
HLA-B27	0.001	0.015	B27 + DRB1*01:16	0.002	0.077
*TPMT* (rs2842934)	0.002	0.026	B27 + DRB1*01:03	0.004	0.115
*IL4* (rs2243250)	0.0025	0.028	HLA-DRB1*04:16	0.007	0.16
HLA-DRB1*13	0.01	0.098	IL4 (rs2243250)	0.007	0.13
*FCGR2A* (rs1801274)	0.011	0.095	BTNL2 (rs3817963)	0.015	0.24
*BTNL2* (rs3817963)	0.017	0.13	*IL17A* (rs2275913)	0.015	0.22
HLA-DRB1*07	0.02	0.14	HLA-DRB1*01:04	0.017	0.22
*STAT4* (rs7574865)	0.025	0.16	*FCGR2A* (rs1801274)	0.026	0.29
*IL17A* (rs2275913)	0.034	0.2	B27 + DRB1*04:16	0.012	0.23
			*STAT4* (rs7574865)	0.068	0.78

The values of alleles and genotypes that remained valid after the Benjamin-Hochberg correction are indicated in gray.

**Table 4:** Genes and loci significantly associated with the clinical characteristics of patients in the study population.

Gene	rs	*p*-Value	Associations with Baseline Value
*DHODH*	rs3213422	0.041	CRP
*MTHFR*	rs180113	0.03	
*PADI4*	rs11203367	0.009	CDAI
		0.012	DAS28
		0.007	HAQ-DI
	rs2240340		
		
		
	rs11203366		
		0.012	
		0.02	
	rs874881	0.014	
		0.017	
		0.016	
		0.003	
		0.043	HAQ-DI
*AMPD1*	rs17602729	0.037	HAQ-DI
*HLA-B27*	-	0.008	Anti-CCP
*IL23R*	rs7539625	0.014	
	rs10889671	0.028	
		0.023	DAS28
		0.005	Anti-CCP
	rs7530511	0.018	HAQ-DI
	rs11465770		
*IL2RB*	rs3218253	0.022	Anti-CCP
*IL6R*	rs2228144	0.043	
*STAT4*	rs7574865	0.037	
*TNFRSF1A*	rs767455	0.022	
	rs1800693	0.01	
*IL2RA*	rs2104286	0.042	RF
*IRAK3*	rs11541076	0.011	
*IL4R*	rs1801275	0.027	
		0.002	DAS28
		0.041	CDAI
*IL4*	rs2243250	0.014	CDAI
		0.015	DAS28
*IL6*	rs2069849	0.032	CDAI

## Data Availability

https://doi.org/10.6084/m9.figshare.14199926.v4 (accessed on 11 March 2021).

## Supplementary Materials

The following are available online at https://www.mdpi.com/article/10.3390/jpm11060469/s1: Table S1: Statistically significant results of an association analysis between SNPs of non-HLA genes and clinical baseline characteristics of patients. The following are available online at https://doi.org/10.6084/m9.figshare.14454594.v1 (accessed on 21 April 2021) and https://doi.org/10.6084/m9.figshare.14199926.v4, IAD177464_185_coverage_summary.
