# Supplementary material for: Genetic Factors of Predisposition and Clinical Characteristics of Rheumatoid Arthritis in Russian Patients
Source: J Pers Med. 2021 May 25;11(6):469. doi: 10.3390/jpm11060469 (PMC8228085; doi:10.3390/jpm11060469)
Supplement: Supplementary file 1 [file jpm-11-00469-s001.zip › jpm-1215361-supplementary.pdf]

Table S1. Statistically significant results of an association analysis between SNPs of non-HLA genes and clinical baseline characteristics of patients.

SNP / Gene (nucleotide substitution)	Genotypes			p-value
**rs3093024 / CCR6 (A>G)**	**A/A**	**A/G**	**G/G**	
Age (years)				0.0019
n	30	60	35	
mean	48.6	54.4	44.9	
SD	12.55	12.37	12.97	
**rs2104286 / IL2RA (T>C)**	**T/T**	**T/C**	**C/C**	
Age (years)				0.0119
n	77	42	6	
mean	52.5	45.6	56.5	
SD	12.66	13.55	6.02	
**rs419598 / IL1RN (T>C)**	**T/T**	**T/C**	**C/C**	
Age (years)				0.0131
n	67	47	11	
mean	52.9	46.0	53.8	
SD	13.12	11.58	15.48	
**rs549908 / IL18 (T>G)**	**T/T**	**T/G**	**G/G**	
Age (years)				0.0269
n	63	56	6	
mean	48.3	51.4	62.7	
SD	12.65	12.93	14.50	
**rs1800629 / TNF (G>A)**	**G/G**	**G/A**	**A/A**	
Age (years)				0.0275
n	104	21	0	
mean	51.5	44.6		
SD	12.99	12.60		
**rs3817963 / BTNL2 (T>C)**	**T/T**	**T/C**	**C/C**	
Age (years)				0.0311
n	60	48	17	
mean	52.5	50.4	43.0	
SD	13.49	13.02	9.69	
**rs1974226 / IL17A (C>T)**	**C/C**	**C/T**	**T/T**	
Age (years)				0.0411
n	89	29	7	
mean	48.8	52.5	60.7	
SD	13.19	11.65	14.13	
**rs2240336 / PADI4 (C>T)**	**C/C**	**C/T**	**T/T**	
Disease duration (years)				0.0038
n	46	62	17	
mean	3.91	8.05	5.91	
Q1, Q3	1.85, 8.35	4.16, 13.07	2.23, 9.97	
**rs4810485 / CD40 (T>G)**	**T/T**	**T/G**	**G/G**	
Disease duration (years)				0.0110
n	7	43	75	
mean	11.16	3.66	6.24	
Q1, Q3	9.97, 13.48	1.45, 8.35	3.16, 11.35	
**rs3817963 / BTNL2 (T>C)**	**T/T**	**T/C**	**C/C**	
Disease duration (years)				0.0131
n	60	48	17	
mean	7.70	3.52	6.71	
Q1, Q3	4.21, 11.65	1.36, 8.30	3.88, 11.03	
**rs3213422 / DHODH (A>C)**	**A/A**	**A/C**	**C/C**	
Disease duration (years)				0.0132
n	27	63	35	
mean	8.35	6.13	4.16	
Q1, Q3	3.66, 13.48	2.75, 11.47	1.10, 7.46	

Table S1. Statistically significant results of an association analysis between SNPs of non-HLA genes and clinical baseline characteristics of patients.

**rs360718 / IL18 (A>C)**	**A/A**	**A/C**	**C/C**	
Disease duration (years)				0.0240
n	69	49	7	
mean	5.17	8.38	1.44	
Q1, Q3	2.50, 8.84	4.16, 13.48	0.73, 13.07	
**rs187238 / IL18 (C>G)**	**C/C**	**C/G**	**G/G**	
Disease duration (years)				0.0244
N	72	48	5	
Mean	5.19	8.55	1.44	
Q1, Q3	2.41, 8.91	4.02, 13.97	1.10, 4.20	
**rs360717 / IL18 (G>A)**	**G/G**	**G/A**	**A/A**	
Disease duration (years)				0.0449
n	72	53 (G/A + A/A)		
Mean	5.19	8.34		
Q1, Q3	2.41, 8.91	3.16, 13.48		
**rs2301888 / PADI4 (G>A)**	**G/G**	**G/A**	**A/A**	
Disease duration (years)				0.0293
n	59	51	15	
Mean	4.20	8.34	5.91	
Q1, Q3	2.32, 8.47	4.52, 13.07	2.23, 10.86	
**rs2476601 / PTPN22 (A>G)**	A/A	A/G	G/G	
Disease duration (years)				0.0414
n	13	32	80	
Mean	3.37	4.68	7.08	
Q1, Q3	2.06, 6.73	1.44, 8.41	3.54, 11.50	
**rs1801133 / MTHFR (G>A)**	**G/G**	**G/A**	**A/A**	
Baseline CRP (mg/mL)				0.0299
n	68	49	8	
mean	2.79	2.68	1.88	
SD	0.848	0.985	0.966	
**rs3213422 / DHODH (A>C)**	**A/A**	**A/C**	**C/C**	
Baseline CRP (mg/mL)				0.0410
n	27	63	35	
Mean	12.0	13.0	20.0	
Q1, Q3	7.0, 22.0	6.0, 24.0	11.0, 41.0	
**rs7530511 / IL23R (T>C)**	**T/T**	**T/C**	**C/C**	
Baseline ACPA (IU/mL)				0.0038
n	3	32	88	
mean	2840.40	626.40	131.50	
Q1, Q3	1176.70, 3018.40	155.50, 1220.05	55.85, 672.30	
Baseline ACPA (IU/mL) n				0.0046
n	35 (TT+TC)		88	
Mean	663.50		131.50	
Q1, Q3	194.70, 1296.90		55.85, 672.30	
**rs7539625 / IL23R (G>A)**	**G/G**	**G/A**	**A/A**	
Baseline ACPA (IU/mL)				0.0140
n	67	50	6	
mean	336.60	250.20	37.30	
Q1, Q3	71.70, 1296.90	70.80, 685.00	1.10, 89.80	
Baseline уровень ACPA (МЕ/мл)				0.0369
n	67	56 (G/A+A/A)		
Mean	336.60	148.05		
Q1, Q3	71.70, 1296.90	37.30, 651.10		
**rs1800693 / TNFRSF1A (T>C)**	**T/T**	**T/C**	**C/C**	
Baseline ACPA (IU/mL)				0.0096

Table S1. Statistically significant results of an association analysis between SNPs of non-HLA genes and clinical baseline characteristics of patients.

N	41	59	23	
Mean	631.10	108.80	323.40	
Q1, Q3	116.20, 1060.10	18.60, 621.70	52.40, 1460.40	
**rs3218253 / IL2RB (G>A)**	**G/G**	**G/A**	**A/A**	
Baseline ACPA (IU/mL) n				0.0215
n	78	40	5	
Mean	239.20	200.05	1376.40	
Q1, Q3	59.30, 759.40	56.70, 834.50	1301.30, 1710.50	
**rs767455 / TNFRSF1A (T>C)**	**T/T**	**T/C**	**C/C**	
Baseline ACPA ACCP (IU/mL) n				0.0215
n	38	56	29	
Mean	407.55	110.65	323.40	
Q1, Q3	116.20, 999.00	11.30, 641.05	61.00, 1185.70	
**rs10889671 / IL23R (A>G)**	**A/A**	**A/G**	**G/G**	
Baseline ACPA (IU/mL) n				0.0277
N	3	32	88	
Mean	2840.40	422.10	151.80	
Q1, Q3	1176.70, 3018.40	80.50, 960.10	60.15, 748.85	
**rs7574865 / STAT4 (T>G)**	**T/T**	**T/G**	**G/G**	
Baseline ACPA (IU/mL)				0.0369
n	8	52	63	
Mean	109.70	376.65	236.70	
Q1, Q3	0.90, 119.20	82.00, 1181.15	22.20, 704.00	
**rs2228144 / IL6R (G>A)**	**G/G**	**G/A + A/A**		
Baseline ACPA (IU/mL)				0.0430
n	81	42		
Mean	381.90	112.05		
Q1, Q3	80.70, 1024.50	19.60, 427.50		
**rs4845374 / IL6R (T>A)**	**T/T**	**T/A + A/A**		
Baseline ACPA (IU/mL)				0.0470
n	79	44		
Mean	381.90	119.25		
Q1, Q3	80.30, 1060.10	20.90, 479.65		
**rs11541076 / IRAK3 (A>T)**	**A/A**	**A/T**	**T/T**	
Baseline RF (IU/mL)				0.0113
n	97	26	2	
Mean	117.0	42.5	24.0	
Q1, Q3	39.0, 334.0	15.0, 119.0	11.0, 37.0	
Baseline RF (IU/mL)				
n	97	28 (A/T + T/T)		0.0040
Mean	117.0	40.5		
Q1, Q3	39.0, 334.0	14.0, 99.5		
**rs1801275 / IL4R (A>G)**	**A/A**	**A/G**	**G/G**	
Baseline RF (IU/mL)				0.0272
n	77	44	4	
Mean	111.0	60.5	339.0	
Q1, Q3	34.0, 321.0	27.0, 165.5	176.5, 475.5	
**rs2104286 / IL2RA (T>C)**	**T/T**	**T/C**	**C/C**	
Baseline RF (IU/mL)				0.0418
n	77	48 (T/C+C/C)		
Mean	117.0	55.0		
Q1, Q3	39.0, 334.0	21.0, 239.5		
**rs11203367 / PADI4 (T>C)**	**T/T**	**T/C**	**C/C**	
Baseline CDAI				0.0086

Table S1. Statistically significant results of an association analysis between SNPs of non-HLA genes and clinical baseline characteristics of patients.

n	27	62	34	
Mean	37.20	40.10	34.95	
Q1, Q3	34.00, 43.40	34.90, 49.00	29.80, 39.50	
**rs2240340 / PADI4 (T>C)**	**T/T**	**T/C**	**C/C**	
Baseline CDAI				0.0115
n	27	61	35	
Mean	37.20	39.40	35.60	
Q1, Q3	34.00, 43.40	34.90, 49.00	29.80, 40.80	
**rs11203366 / PADI4 (G>A)**	**G/G**	**G/A**	**A/A**	
Исходное значение CDAI				0.0171
n	26	62	35	
Медиана	36.80	39.10	35.60	
Q1, Q3	34.00, 43.40	34.90, 49.00	29.80, 41.20	
**rs2243250 / IL4 (C>T)**	**C/C**	**C/T**	**T/T**	
Baseline CDAI				0.0142
n	69	45	9	
Mean	35.60	39.30	41.16	
Q1, Q3	31.63, 42.40	35.20, 47.40	35.90, 50.10	
Baseline CDAI				0.0048
n	69	54 (C/T+T/T)		
Mean	35.60	40.10		
Q1, Q3	31.63, 42.40	35.30, 47.50		
**rs2069849 / IL6 (C>T)**	**C/C**	**C/T**	**T/T**	
Baseline CDAI				0.0317
n	120	3	0	
Mean	37.95	30.60		
Q1, Q3	33.45, 45.65	25.50, 33.80		
Baseline CDAI				0.0324
n	120	3 (C/T + T/T)		
Mean	37.95	30.60		
Q1, Q3	33.45, 45.65	25.50, 33.80		
**rs1801275 / IL4R (A>G)**	**A/A**	**A/G**	**G/G**	
Baseline CDAI				0.0408
n	75	44	4	
Mean	36.30	38.60	51.25	
Q1, Q3	32.80, 43.10	33.45, 46.80	44.45, 62.75	
**rs10889671 / IL23R (A>G)**	**A/A**	**A/G**	**G/G**	
Baseline CDAI				0.0460
n	88	35 (A/G + A/A)		
Mean	38.75	35.30		
Q1, Q3	34.15, 47.20	32.80, 39.40		
**rs1801275 / IL4R (A>G)**	**A/A**	**A/G**	**G/G**	
Baseline DAS28- CRP				0.0017
n	75	44	4	
Mean	5.94	5.84	7.02	
SD	0.594	0.642	0.733	
**rs11203367 / PADI4 (T>C)**	**T/T**	**T/C**	**C/C**	
Baseline DAS28- CRP				0.0123
n	27	62	34	
Mean	5.84	6.10	5.72	
CO	0.593	0.670	0.560	
**rs2243250 / IL4 (C>T)**	**C/C**	**C/T**	**T/T**	
Baseline DAS28- CRP				0.0152
n	69	45	9	
Mean	5.79	6.10	6.23	

Table S1. Statistically significant results of an association analysis between SNPs of non-HLA genes and clinical baseline characteristics of patients.

SD	0.630	0.604	0.713	
Baseline DAS28- CRP				0.0045
n	69	54 (C/T+T/T)		
Mean	5.79	6.12		
CO	0.630	0.618		
**rs11203366 / PADI4 (G>A)**	**G/G**	**G/A**	**A/A**	
Baseline DAS28- CRP				0.0158
n	26	62	35	
Mean	5.84	6.10	5.73	
SD	0.605	0.672	0.555	
**rs2240340 / PADI4 (T>C)**	**T/T**	**T/C**	**C/C**	
Baseline DAS28- CRP				0.0207
n	27	61	35	
Mean	5.84	6.10	5.74	
SD	0.593	0.674	0.567	
**rs10889671 / IL23R (A>G)**	**A/A**	**A/G**	**G/G**	
Baseline DAS28- CRP				0.0233
n	88	35 (A/G + A/A)		
Mean	6.01	5.76		
SD	0.693	0.461		
**rs2069849 / IL6 (C>T)**	**C/C**	**C/T**	**T/T**	
Baseline DAS28- CRP				0.0463
n	120	3	0	
Mean	5.96	5.21		
SD	0.640	0.196		
**rs11203366 / PADI4 (G>A)**	**G/G**	**G/A**	**A/A**	
Baseline HAQ-DI				0.0025
n	26	62	35	
Mean	1.5096	1.8407	1.5536	
SD	0.6214	0.4573	0.3898	
**rs11203367 / PADI4 (T>C)**	**T/T**	**T/C**	**C/C**	
Baseline HAQ-DI				0.0066
n	27	62	34	
Среднее	1.5324	1.8286	1.5588	
SD	0.6207	0.4595	0.3944	
**rs2240340 / PADI4 (T>C)**	**T/T**	**T/C**	**C/C**	
Baseline HAQ-DI				0.0137
n	27	61	35	
Mean	1.5324	1.8197	1.5821	
SD	0.6207	0.4579	0.4123	
**rs17602729 / AMPD1 (G>A)**	**G/G**	**G/A**	**A/A**	
Baseline HAQ-DI				0.0368
n	89	28	6	
Mean	1.7107	1.5357	2.0833	
SD	0.4521	0.6188	0.3227	
**rs874881 / PADI4 (G>C)**	**G/G**	**G/C**	**C/C**	
Baseline HAQ-DI				0.0428
n	33	62	28	
Mean	1.5871	1.8004	1.5625	
SD	0.5867	0.4883	0.3527	

CO= Standard deviation; CRP = C-reactive protein; DAS28 = Disease Activity Score 28-joint Count; CDAI = Clinical Disease Activity Index; HAQ-DI = Health Assessment Questionnaire – Disability Index; ACPA – anti -citrullinated peptide antibodies. RF- rheumatoid factor. n: the number of patients in the corresponding category or the number of valid observations.
Q1 (Q3): 1 (3) quartile.
